# Meteo and Hydrodynamic Measurements to Detect Physical Processes in Confined Shallow Seas

**DOI:** 10.3390/s18010280

**Published:** 2018-01-18

**Authors:** Francesca De Serio, Michele Mossa

**Affiliations:** Department of Civil, Environmental, Land, Building Engineering and Chemistry (DICATECh), Polytechnic University of Bari, via Orabona 4, Bari 70125, Italy; michele.mossa@poliba.it

**Keywords:** hydrodynamic patterns, field measurements, monitoring stations, semi-enclosed basin

## Abstract

Coastal sites with typical lagoon features are extremely vulnerable, often suffering from scarce circulation. Especially in the case of shallow basins subjected to strong anthropization and urban discharges, it is fundamental to monitor their hydrodynamics and water quality. The proper detection of events by high performance sensors and appropriate analysis of sensor signals has proved to be a necessary tool for local authorities and stakeholders, leading to early warning and preventive measures against environmental degradation and related hazards. At the same time, assessed datasets are not only essential to deepen the knowledge of the physical processes in the target basin, but are also necessary to calibrate and validate modelling systems providing forecasts. The present paper aims to show how long-term and continuous recordings of meteorological and hydrodynamic data, collected in a semi-enclosed sea, can be managed to rapidly provide fundamental insights on its hydrodynamic structure. The acquired signals have been analyzed in time domain, processed and finally, correlated. The adopted method is simple, feasible and easily replicable. Even if the results are site-dependent, the procedure is generic, and depends on having good quality available data. To show how this might be employed, a case study is examined. In fact, it has been applied to a coastal system, located in Southern Italy, where two monitoring stations are placed in two interconnected basins. The inferred results show that the system is not wind dominated, and that the annual trends in the wind regime, wave spreading and current circulation are not independent, but rather reiterate. These deductions are of great interest as a predictive perspective and for numerical modelling.

## 1. Introduction

Two of the primary applications for new sensor technologies are environment health monitoring and control systems. Observing, controlling, and/or predicting dangerous events, can provide advanced warning to allow for repair or intervention. Coastline erosion as well as diffusion and dispersion of polluting tracers are hazardous phenomena, especially for urban sites facing the sea, thus they need to be strictly monitored. With the primary goals being to enhance safety and to adapt to climate changes, the need for effective control of the coastal hydrodynamics is clear. In fact, all of the above-mentioned processes are strictly linked to currents’ magnitudes and directions [1,2,3,4]. Shallow and confined seas, like lagoons, require even greater monitoring action, being mainly subjected to water stagnation and long water renewal times. These factors can cause sediment trapping and accumulation of polluting substances, thus damaging environmental health. Therefore, coastal management plans and in situ decision-making should include actions to guarantee thorough knowledge of hydrodynamic processes [5,6,7]. Generally, numerical models are preferred in this scope, because they allow us to reproduce and predict marine physical phenomena in a relatively short time, with accuracy and moderate costs [8,9,10]. Predictive operational oceanography commonly uses models covering regional, sub-regional and shelf-coastal scales. To study local scales, with a resolution of a few hundred meters, multiscale modelling systems, based on a multiple-nesting approach, have been implemented lately [8,9,11,12]. In all cases, they require calibration and validation with measured data. Further, the quantity, quality and duration of the observations determine the accuracy of model outputs [13,14].

In addition, high quality field data—continuously recorded for long periods and with high resolution over time—allow us to identify some typical patterns and recurring trends in the investigated site, even without resorting to modelling. Therefore, they should not simply be stored in repositories. The data driven approach has been in use in hydrological studies for nearly three decades [15]. On the contrary, in ocean and coastal communities, it started to be considered recently, with the application of statistical analysis techniques, when it was recognized that many coastal data sets display coherent patterns of temporal behavior which characterize physical processes [13,16,17]. Further, such information could be extrapolated to provide a prediction of future coastal states [13,14]. Increasingly sophisticated statistical methods have been used in this approach [16,17,18]. Nonetheless, a greater complexity in methods of analysis is not a guarantee of more accurate and reliable descriptions of the processes, which, on the contrary, mainly depend on the quality of available data.

**Figure 1:**
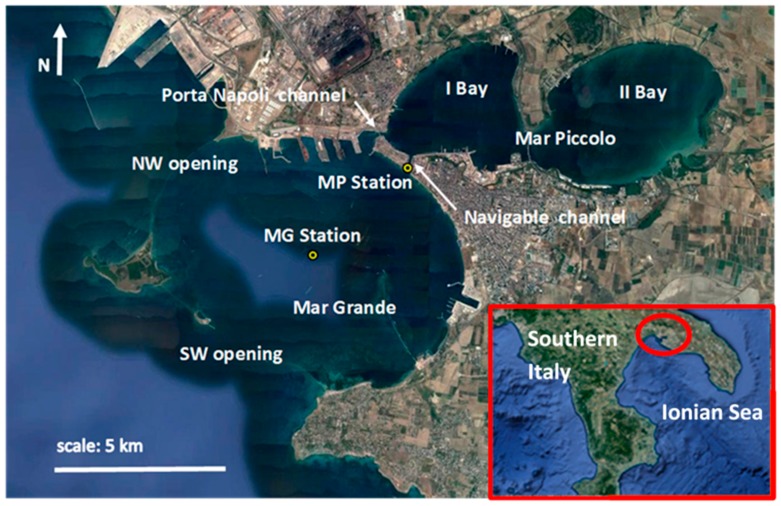
Map of Mar Grande (MG) and Mar Piccolo (MP) coastal system, with the locations of the two monitoring stations, MG and MP. Google Earth source.

For this reason, the present paper aims to show that sensors can be used to gather a wealth of information from the examined process, which can be used to perform challenging tasks and specifically, improve operational efficiency. In fact, the sole analysis in time domain of an interannual series of wind, wave and current data, even if simple, allows us to deduce: (1) spatial and temporal variability of wind, wave and current spreading; (2) recurring patterns in the examined basin; (3) correlations among the examined parameters; and (4) water mass transport and exchange fluxes along connecting channels. As previously stated, the fundamental precondition to achieving this aim is having high quality data (i.e., we use data with high spatial and temporal resolution, continuously sampled and simultaneously assessed at the same position). This means that fundamental requirements for the temporal length of the acquired signals, acquisition frequencies, absence of gaps in the time series and simultaneous measurements of more parameters in the same location, must be respected [13,19]. Specifically, our procedure is applied to a study area in Southern Italy, in the inner Ionian Sea (Figure 1), where two monitoring stations are located. Accordingly, the deduced results are site specific, but the adopted procedure is general and replicable. Therefore, sensing technology remains a fundamental support for coastal management, planning and audit, providing high quality data, which is necessary to have knowledge of, especially in vulnerable regions.

## 2. Materials and Methods: Set Up and Equipment

### 2.1. Study Site

The area in question is in Southern Italy and is composed of two basins—an inner one called Mar Piccolo and an external one named Mar Grande (Figure 1). Mar Piccolo, whose total surface is around 21.7 km^2^, is formed by two bays, while Mar Grande, with a surface of around 35 km^2^, has a typical round shape. In the I Bay of Mar Piccolo, the maximum depth is around 15 m, while in the II Bay, it is around 10 m. The I Bay is joined to Mar Grande by means of two channels: an artificial channel (the Navigable Channel), and a natural channel (the Porta Napoli Channel) (Figure 1). The Navigable Channel is 58 m wide, 375 m long and 14 m deep, while the Porta Napoli Channel is 150 m wide and 2.5 m deep. Considering these dimensions and the complex topography, the hydrodynamic patterns in this coastal system can be properly studied only on a local scale. As shown in Figure 1, the Mar Grande is connected to the open sea by means of two openings, located respectively along its Northwestern and Southwestern boundaries.

This target area is highly vulnerable, because it is exposed to a strong human pressure, to urban and industrial discharges and to intense naval traffic [19,20,21]. For all these reasons, at present, it is enclosed on the so-called SIN (site of national interest) list and is under the control of the Special Commissioner, appointed by the Italian Government, to evaluate and put in place urgent measures for the remediation and environmental requalification of Taranto city.

### 2.2. Instrumentations Settlement, System Configurations and Data Transmission

**Figure 2:**
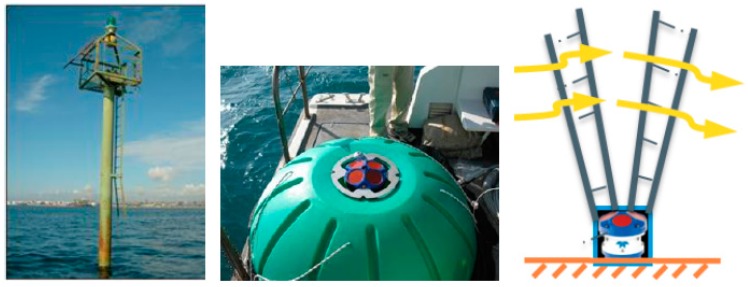
Station in Mar Grande (MG): details of the meteo sensor (**left**) and of the Acoustic Doppler Current Profiler (ADCP) sensor (**central**). A sketch of the ADCP functioning is also shown (**right**).

In December 2013, a meteo-oceanographic station (Mar Grande, MG station) was fixed in the Mar Grande basin (Figure 1), at the geographical coordinates, 40°27.6′ N and 17°12.9′ E [20,21]. The local depth in this station is, on average, equal to 23.25 m. In addition to other equipment, a bottom-mounted Acoustic Doppler Current Profiler (ADCP), a multidirectional wave array and a weather station were installed in this station (Figure 2).

In detail, the weather system records speed and wind direction at 1.5 m above the sea surface by means of an ultrasonic sensor. The hourly-averaged values of wind speed and direction are given with an accuracy of ±2% of the velocity value and ±3° of the direction.

The ADCP measures the 3D velocity of currents along the vertical axis. It uses a Janus configuration, consisting of four acoustic beams, paired in orthogonal planes, where each beam is inclined at a fixed angle of 20° to the vertical axis. This configuration allows the measurement of the error velocity and thus, a quality check of the recorded datum. The ADCP (Figure 2) is bottom-mounted, upward-facing and has a pressure sensor for measuring mean water depth. The transducer head is at 0.5 m above the seafloor. Velocities are sampled along the water column with an 0.5 m vertical bin resolution and a 1.60 m blanking distance. Therefore, the water column is investigated from a distance from the sea bottom of z = 2.1 m, up to the most superficial bin not biased by waves. The surface layer, with a thickness, on average, equal to 2.0 m, is excluded from the analysis, to filter out possible noise in the measurements as well as the wave contribution to currents. Mean current velocity profiles are collected continuously, at 1-h intervals, using an average of 60 measurements, acquired every 10 s [4]. In this way, hourly-averaged velocity components along the water column are available [22].

In May 2014, the Mar Piccolo station (MP) was also placed in the target area. Specifically, it was installed in the Navigable Channel, at the geographical coordinates, 40.473° N and 17.235° E (Figure 1). It is equipped with a bottom-mounted ADCP and a wave array. The local depth at this station is, on average, 13.7 m. Also in this case, considering the ADCP size and its blanking distance, the current velocities are assessed along the vertical axis, starting from z = 2.1 m from the sea bed, at constant intervals of 0.5 m, up to the most detectable unbiased bin, at z = 12.6 m. The acoustic frequency of both the installed ADCPs is 600 KHz and their velocity accuracy is 0.3% of the water velocity ±0.003 m/s.

In both MG and MP stations, the ADCP measures the component of velocity projected along the beam axis, averaged over a range cell. Since the mean current is assumed to be horizontally uniform over the beams, its components can be recovered as linear combinations of the measured along-beam velocities. The situation regarding wave measurements is more complicated, since, at any instant, the wave velocities vary spatially across the array. As a result, apart from very long waves that remain coherent during their passage from one beam to another, it is not possible to separate the horizontal and vertical wave velocity components. However, the wave field is statistically steady in time, and homogeneous in space, and therefore cross-spectra between velocities measured at various range cells (either beam to beam or along each beam) contain information about wave direction [10]. In other words, each depth cell of the ADCP can be considered an independent sensor that makes a measurement of one component of the wave field velocity. The ensemble of depth cells along the four beams constitutes an array of sensors, from which magnitude and directional information about the wave field can be determined. Therefore, this instrument is able to provide a multidirectional wave spectrum.

**Figure 3:**
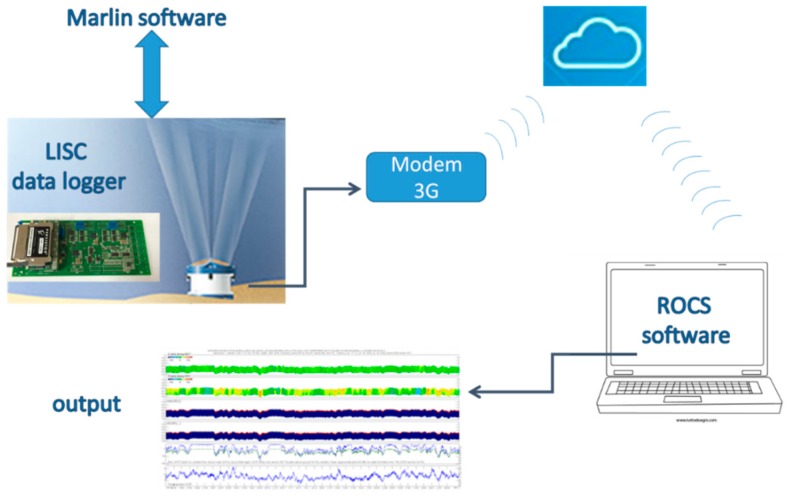
Sketch of data transmission, from sensors to managing remote systems.

Both stations—MG and MP—work with analogous configurations [13,19]. The sensors are all connected to a datalogger (named LISC), which is an autonomous data acquisition unit, able to acquire data from 12 serial ports and 16 analogic channels. It allows remote control and data download. The data from the ADCPs and the multidirectional wave meters are processed in real time by this datalogger. This operation is heavy in computing terms and requires high-energy consumption. Therefore, it is managed by the software, MARLIN, installed in a proper device, which connects to the LISC at the end of the measurements for a short time window, just necessary to get the measured data and send back the processed data. The communication with remote systems is possible by means of a cellular modem 3G, connected to the datalogger through a serial port and provided with a stack TCP/IP to send data on the web cloud. In this way, remote systems can be reached, and communication from the web to the devices is possible, managed by proper software (named ROCS). This allows to more LISC dataloggers to be called, either automatically or manually, to download acquired data, and to check or change acquisition parameters. The ROCS core is a relational database, containing configurations and data from all of the managed stations. A ROCS module further allows the export of binary raw data to ascii files. The whole system is managed and controlled by the research unit of the LIC (Laboratory of Coastal Engineering) of the Polytechnic University, in Bari. In Figure 3, a sketch of data transmission is plotted as an example.

After a first period of testing, both stations (MG and MP) started to collect a large amount of data, for about three years. In the present work, wind, waves and currents are investigated, from January 2014 up to December 2016 for station MG, and from January 2015 up to December 2016 for station MP. All these data are vector data, sampled with an hourly frequency. Referring to currents, the two velocity components in the horizontal plane, at all of the investigated depths, are considered in this work.

## 3. Data Analysis and Discussion of Results

For each investigated year, a sequence of analyses has been carried out on the data records of wind, wave heights and sea current velocity components. Firstly, recorded raw data from stations MG and MP have been processed, converting them from binary files to editable files. Successively, filters have been applied to remove bias, and data have been stored in monthly tables, selecting hourly values for each detected parameter. After this, each annual time series has been processed in the following way: based on the maximum amplitude of the recorded signal, an adequate number of classes has been fixed, each one with a specific amplitude. Data have been grouped in these classes, according to their values, and consequently, the frequency of occurrence of each value has been derived. In order to take into account the fact that all the managed data embeds double information (i.e., a magnitude and a direction), the best solution to display the occurrence of these events is a polar plot representation. Some of the data assessed in the years 2014 and 2015 and analyzed in previous works [13,19] are presented afterwards, for comparison with the data from the year 2016.

### 3.1. Data Analysis in MG Station

#### 3.1.1. Winds and Waves

**Figure 4:**
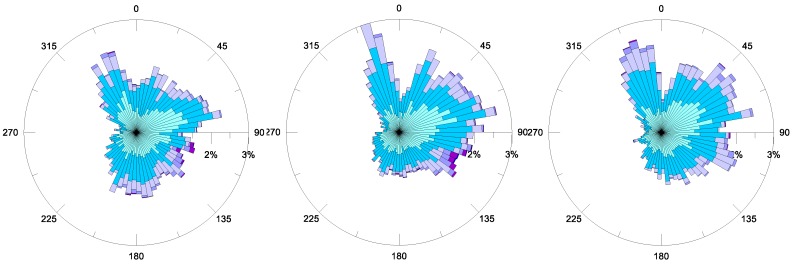
Annual winds recorded at station MG (m/s) for the years 2014 (**left**), 2015 (**central**) and 2016 (**right**). The incoming direction is shown.

Figure 4 exhibits the annual polar plots of the wind field from the MG station, respectively measured in the years 2014, 2015 and 2016. In all cases, winds from the NNW direction are the most frequent, with maximal intensities generally around 9 m/s. Winds spanning clockwise from NNE to E have moderate velocities (6–9 m/s) and are moderately frequent. Along these directions, both moderate and low winds (3–6 m/s) become more frequent in 2015 and 2016, with respect to the year 2014. The most intense winds are typically Southern and above all, Sirocco winds (i.e., blowing from the SE), with intensities in the range of 12–15 m/s, even if they are less frequent. It is evident that the wind distribution has a recursive annual behavior, thus, each year confirms what was already observed in the previous one, at the same station [13,19]. It is worth noting that winds contributing to wave formation are winds coming from the open sea, namely, those blowing within the limits of the geographical fetch. Due to the geographical position of the station, near the center of the Mar Grande basin (Figure 1), winds spanning from S to NW should be the most effective for wave generation. Nevertheless, the round and confined shape of the Mar Grande basin, whose boundaries are interrupted by two openings—in the NW and SW—induces very limited fetches. Therefore, it can be assumed that wind waves rarely occur in this coastal basin because of short fetches and dominant inland winds. On the contrary, the topography of the basin makes it prevalently dominated by swell waves, generated outside the Mar Grande, which propagate inside it, in particular, passing through the Southwestern opening.

**Figure 5:**
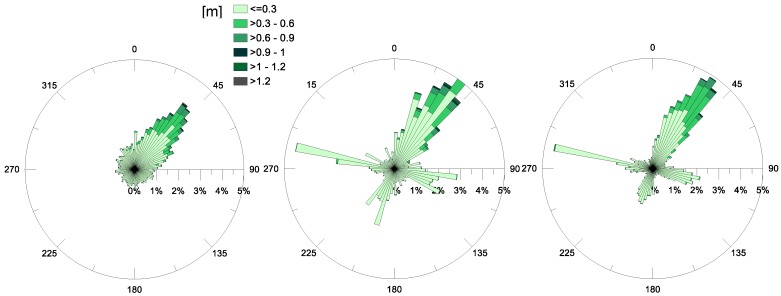
Annual significant wave heights *Hs* (m) recorded at station MG, for the years 2014 (**left**), 2015 (**central**) and 2016 (**right**). The direction of propagation is shown.

This observation endorses the results highlighted by [11], and is proven by the investigation of the annual polar plots for the significant wave heights (*Hs*) recorded at the MG station and presented in Figure 5. For all of the observed years, the prevalent direction of propagation of these waves, calculated to be a third of the highest waves recorded in the time series, is NE. This means that the significant wave heights have an incoming direction from SW, consistent with the principal opening in the Mar Grande border (Figure 1). Even the highest values of *Hs* ≥ 1 m are observed along this direction.

To thoroughly confirm that the wave regime in this basin is not wind dominated, the relation between wind speed, wave height, and wave period has been examined. In a wind-generated ocean wave system, the three parameters—*Hs*, peak period (*Tp*) and wind speed (*Uw*)—are closely related in the following equation, established through theoretical analysis of wave dynamics, and confirmed with several groups of experimental data [23]:

(1) $$\frac{U_{w}}{gT_{p}}=0.048{\left(\frac{U_{w}{}^{2}}{gH_{s}}\right)}^{0.67}$$

where *g* is the gravitational acceleration.

**Figure 6:**
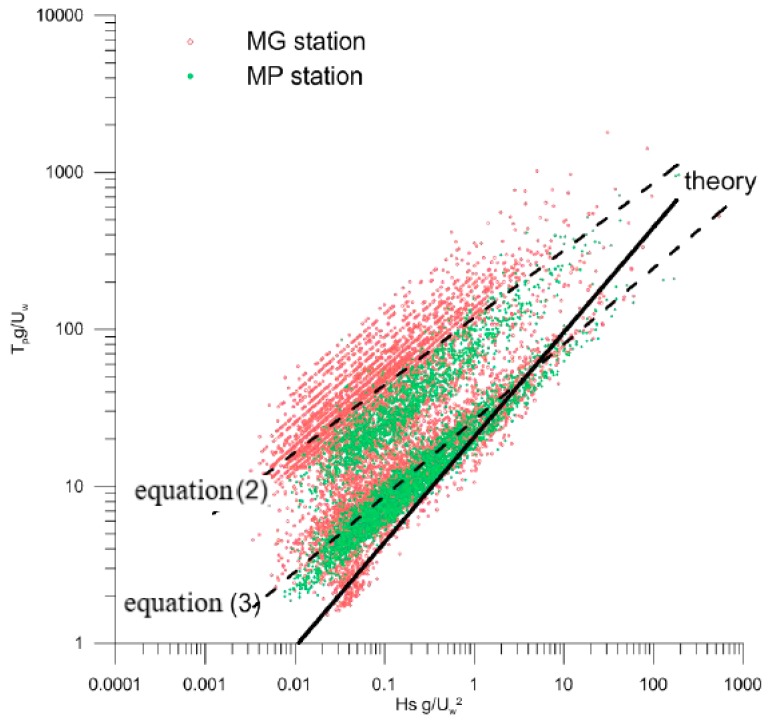
Relationships between wind intensity and significant wave height and period. The solid line is the theoretical wind–sea relationship of Equation (1). Dotted lines represent the newly-derived Equations (2) and (3).

Therefore, the measured *Hs* and *Tp* have been normalized, respectively, with *g/Uw*^2^ and *g/Uw*. Their scatter plot is drawn in a log scale, in Figure 6, where all the recorded data are included. It is evident that they tend to collapse in two different lines (dotted lines), far from the fully-developed theoretical wind-sea curve (solid line). The wind-sea condition represents a very small fraction of the total wave regime in the study region—about 6% of the total data. Mostly, wave data generally have *Hs*/(*gTp*^2^) < 0.0025, and are best fitted by the two following relations:

(2) $$\frac{U_{w}}{gT_{p}}=0.039{\left(\frac{U_{w}{}^{2}}{gH_{s}}\right)}^{0.49}$$

(3) $$\frac{U_{w}}{gT_{p}}=0.0088{\left(\frac{U_{w}{}^{2}}{gH_{s}}\right)}^{0.43}$$

with regression coefficients equal to *R*^2^ = 0.84 and 0.70, respectively.

Therefore, these new proposed expressions describe swells, with different coefficients depending on the values of *Hs* and *Tp*. Because they are deduced from a great amount of experimental data, and in general conditions, they could also be extended to waves matching similar heights and periods. Consequently, the condition *Tp* < 10 s, which sometimes used as a crude method to individuate wind waves and separate them from swells [24], should be considered not exhaustive.

#### 3.1.2. Currents

**Figure 7:**
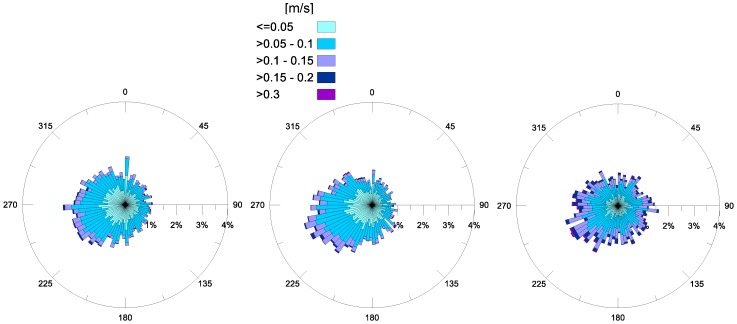
Annual surface horizontal currents (in m/s) at station MG, for the years 2014 (**left**), 2015 (**central**) and 2016 (**right**). The direction of propagation is shown.

The annual pattern of the surface currents, recorded in 2016, confirms the observations of both years 2014 and 2015 (Figure 7). This highlights that superficial currents predominantly propagate in the second and third quadrant (from north to south), with similar annual frequencies for each year. However, in the year 2016, more intense currents (values 0.1–0.2 m/s) were recorded at the MG station. A comparison of Figure 7 with the annual winds shown in Figure 4, highlights that winds blowing from land do not have a direct effect on the origin of sea waves (Figure 5), rather, they seem to drive surface current, whose direction of propagation is consistent with incoming winds, spanning from NE to SE (Figure 4).

**Figure 8:**
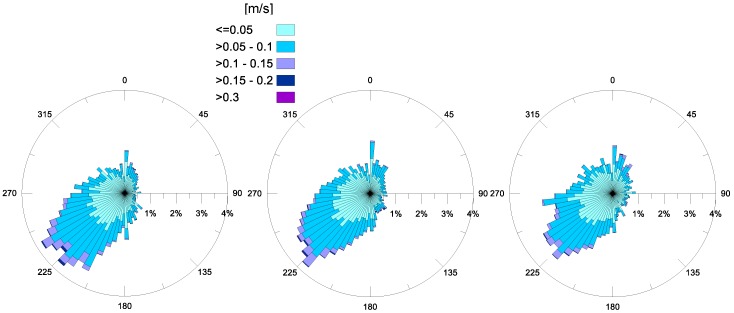
Annual bottom horizontal currents (in m/s) at station MG, for the years 2014 (**left**), 2015 (**central**) and 2016 (**right**). The direction of propagation is shown.

Figure 8 depicts the distribution of the annual bottom currents, varying with years. The most frequent direction of current propagation near the sea bed is the same for all of the three examined years. In fact, currents appear to have a preferred direction and converge towards the SW opening (Figure 1), thus resulting in topographical control.

Relating the intensities of surface currents (Figure 7) and bottom currents (Figure 8), higher values (0.1–0.2 m/s) are always noted near the surface, rather than near the bed (0.05–0.1 m/s). Thus, the energy transferred from wind to surface currents, by means of shear stress, is partially lost along the water column, and the principal driving mechanism at the bottom seems to be the local topography morphology.

### 3.2. Data Analysis in MP Station

**Figure 9:**
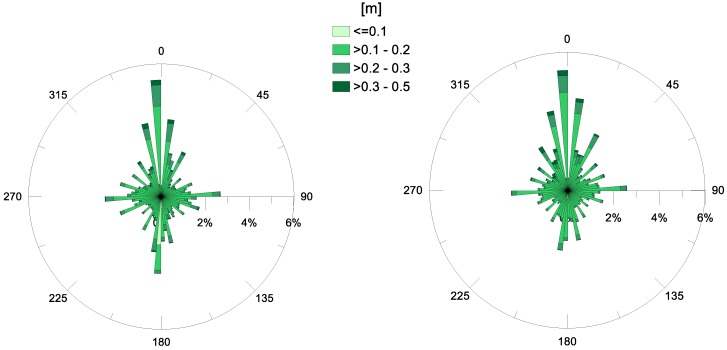
Annual significant wave heights (in m) at station MP, for the years 2015 (**left**) and 2016 (**right**). The direction of propagation is shown.

The analysis carried out for the waves and currents at the MG station has also been applied to the data from the MP station, in the Navigable Channel. The polar plots of the annual *Hs* values are displayed in Figure 9, for years 2015 and 2106. Specifically, Figure 9 highlights that, in both years examined, the most frequently-observed direction of propagation in the channel was north, which is expected to be due to the topography and orientation of the channel itself. In fact, the handmade Navigable Channel has a fairly regular shape, and its longitudinal axis is fairly aligned along the N–S direction (12° clockwise rotated from N, see Figure 1). For this reason, the waves that have already spread through the Mar Grande basin finally enter the channel from the south and propagate inside. The predominant direction of wave propagation is S–N, i.e., confined by the shape of the channel itself. Specifically, the monthly analysis reveals that the direction of significant waves is slightly variable in the months when the heights are generally low, such as June and July. On the contrary, it settles along the longitudinal axis of the Navigable Channel when higher heights are observed (September, October, November).

The smoothing effect, played by the Mar Grande, on waves which are diffracted and refracted, is also evident. In fact, the analysis of Figure 9 shows that values of wave heights (*Hs*) at the MP station are generally low and maximal *Hs* values are around 0.5 m. All previous observations allow us to deduce that the swell waves generated outside the Mar Grande enter it from its SW opening and propagate throughout (Figure 5), experiencing diffraction and refraction effects. Finally, they reach the Navigable Channel, but with heights reduced by about 50% in amplitude and continue travelling towards the Mar Piccolo, confined by the channel lateral boundaries.

**Figure 10:**
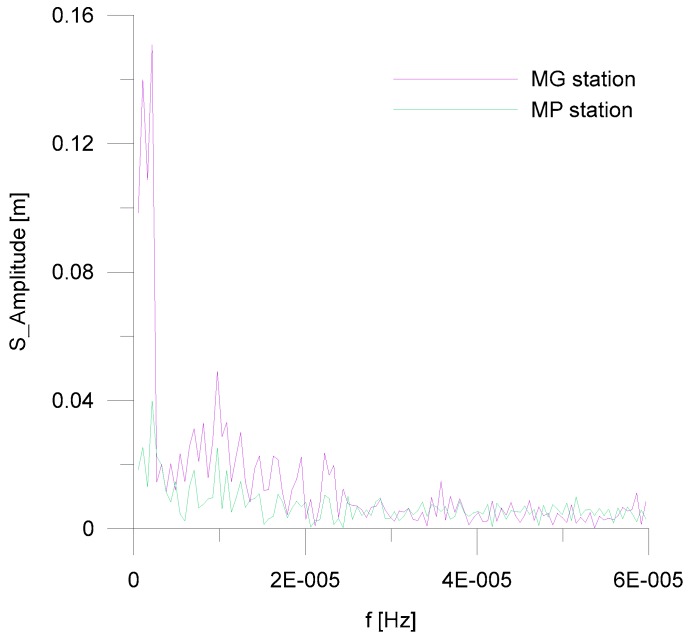
Amplitude spectra of the significant wave heights, recorded at MG and MP stations during November 2015.

It is worth noting that a correlation of the significant wave heights between the two stations, MG and MP, is always visible, in both the time and frequency domains. The analysis of the time series of the measured *Hs* highlights that a time lapse of 2 to 3 h occurs between the waves measured at the MG station and the same waves measured at the MP station. The amplitude spectra of the significant wave heights, calculated for each examined month, show the same frequency peaks for the waves at both stations (MG and MP). Furthermore, the amplitudes at corresponding frequencies are higher at the MG station and lower at the MP station. As an example, Figure 10, for the month of November 2015, is shown.

**Figure 11:**
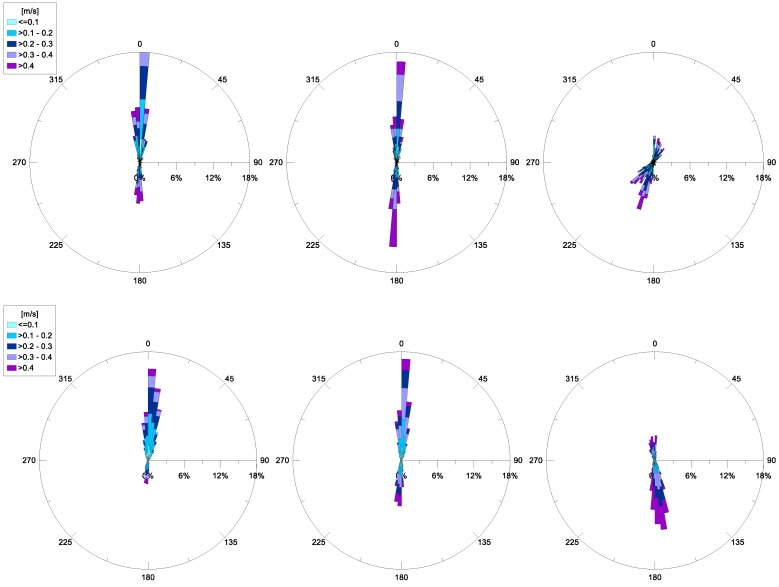
Annual current velocity (m/s) recorded at station MP, at the bottom (**left**), at an intermediate depth (**central**) and near the surface (**right**). The top line shows 2015 data and the bottom line shows 2016 data.

Regarding the horizontal currents at the MP station, their hourly-averaged values have been examined for each month. For the years 2015 and 2016, polar diagrams of these recordings are available. Specifically, current trends at some selected depths (representative of a near-bottom layer, intermediate layer and surface layer) are depicted in Figure 11. In detail, near the bottom (~11 m from the surface) the prevailing current is inflowing, from Mar Grande towards the Mar Piccolo basin, with a dominant direction converging to the longitudinal channel axis. Its most frequent intensities are in the range, 0.05–0.4 m/s. At an intermediate depth (~6 m from the surface) this inflow is still present, but also an intense outflow towards the Mar Grande begins to appear, with similar frequencies. Approaching the surface, a dominant outflowing current, from the Mar Piccolo towards the Mar Grande is recorded, with the highest intensities (even greater than 0.4 m/s).

This vertical distribution for the velocity in the channel highlights a fundamental aspect—in the examined period, a double circulation occurred in the channel, with inflowing currents towards the Mar Piccolo basin in deeper layers and outflowing currents in the most superficial ones. The flux inversion along the vertical axis takes place at an intermediate depth. This trend is recursive, and a thorough analysis has confirmed that this behavior steadily persists in the station, even on a monthly temporal scale. This behavior also proves the numerical results obtained by previous studies, such as [25,26].

A first insight into a flux balance between inflows and outflows, referring to monthly-averaged values, allows us to deduce that on annual average, the inflowing rate prevails over the outflowing one, for the years 2015 and 2016, again endorsing the numerical findings of [25,26], and confirming that the Navigable Channel is the main way through which sea water enters the Mar Piccolo.

## 4. Conclusions

The present study examined a large collection of meteorological and hydrodynamic parameters, recorded by two continuous monitoring systems, located in Mar Grande and Mar Piccolo. Continuous, high quality, inter-annual data series (years 2014–2016) have been analyzed with a simple and repeatable procedure. The main findings allowed us to identify recurrent and typical behaviors and trends in the basins and can be summed up in the following way.

The annual wind distributions always show prevalent winds, blowing from the NNW, which are also moderately intense, while the most intense ones are from the SE. The comparison between the polar plots of winds and significant wave heights at the MG station reveals that the waves in the Mar Grande basin are not wind-generated, but rather, are swells entering from its SW opening. This qualitative result was also proven by checking the validity of the fully-developed wind–wave equation. On the contrary, wind seems to drive surface circulation in the same basin. For all of the examined years, the bottom currents have a quite stationary trend—they converge towards the SW opening of the Mar Grande border, which therefore represents a fundamental topographical element. In fact, it controls both incoming waves and propagating bottom currents.

The wave data have also been examined at the MP station, in the Navigable Channel. They have a direction of propagation, naturally obliged to follow the longitudinal axis of the channel. The significant wave heights are reduced by about 50%, with respect to the values recorded at the MG station, because of diffraction and refraction effects.

For each investigated year, the vertical profiles of the current data measured in MP station highlight the presence of a steady, double circulation in the channel. An inflowing flux from Mar Grande towards the Mar Piccolo basin occurs in deeper layers and an outflowing flux is recorded towards the Mar Grande in the most superficial layers. This exchange mechanism between the two basins confirms previous numerical modelling outputs [25,26]. Even an annually-averaged estimate of the flow rates shows that the inflowing rate prevails over the outflowing one, again endorsing numerical findings and confirming that the Navigable Channel is the main way through which seawater enters the Mar Piccolo basin.

All the results derived in this study display how we can simply, but adequately, describe the state and the evolutionary trends of wind, wave and current interactions in any investigated basin, where typical behaviors are expected to recur. In fact, we have shown that immediate and simple procedures are able to transform high-quality field measurements into useful information for potential stakeholders [10]. In this context, the quantity, quality and duration of observations are extremely valuable.
